# Novelty Recognition: Fish Species Classification via Open-Set Recognition

**DOI:** 10.3390/s25051570

**Published:** 2025-03-04

**Authors:** Manuel Córdova, Ricardo da Silva Torres, Aloysius van Helmond, Gert Kootstra

**Affiliations:** 1 Agricultural Biosystems Engineering Group, Wageningen University and Research, 6700 AA Wageningen, The Netherlands; gert.kootstra@wur.nl; 2 Artificial Intelligence Group, Wageningen University and Research, 6708 PB Wageningen, The Netherlands; ricardo.dasilvatorres@wur.nl; 3 Wageningen Marine Research, Wageningen University and Research, 1970 AB IJmuiden, The Netherlands; edwin.vanhelmond@wur.nl

**Keywords:** novelty detection, fish classification, open-set recognition, computer vision

## Abstract

To support the sustainable use of marine resources, regulations have been proposed to reduce fish discards focusing on the registration of all listed species. To comply with such regulations, computer vision methods have been developed. Nevertheless, current approaches are constrained by their closed-set nature, where they are designed only to recognize fish species that were present during training. In the real world, however, samples of unknown fish species may appear in different fishing regions or seasons, requiring fish classification to be treated as an open-set problem. This work focuses on the assessment of open-set recognition to automate the registration process of fish. The state-of-the-art Multiple Gaussian Prototype Learning (MGPL) was compared with the simple yet powerful Open-Set Nearest Neighbor (OSNN) and the Probability of Inclusion Support Vector Machine (PISVM). For the experiments, the Fish Detection and Weight Estimation dataset, containing images of 2216 fish instances from nine species, was used. Experimental results demonstrated that OSNN and PISVM outperformed MGPL in both recognizing known and unknown species. OSNN achieved the best results when classifying samples as either one of the known species or as an unknown species with an F1-macro of 0.79±0.05 and an AUROC score of 0.92±0.01 surpassing PISVM by 0.05 and 0.03 , respectively.

## 1. Introduction

Fisheries contribute significantly to global food production, providing an important source of human nutrition around the world [1]. In addition to their role in feeding populations, fisheries are essential to the economy of coastal communities. On the other hand, one of the main concerns is that the sustainability of fisheries is increasingly threatened by overfishing [2]. Management of fishing activities is crucial to ensure that fish are harvested responsibly and remain viable for future generations. Effective management should help balance economic needs with conservation efforts. Regulations have been adopted to increase transparency and promote more sustainable use of fish resources. For instance, the Landing Obligation was introduced by the European Union, which requires fishing vessels to land and register the complete catch, including unwanted fish, which would previously have been discarded (Article 15 of Regulation (EU) No 1380/2013, available at http://data.europa.eu/eli/reg/2013/1380/oj (accessed on 28 February 2025)). However, bringing discards to the harbor not only creates an overload of work for fishers but also impacts their economic benefits, as a large portion of space on the fishing vessels must be used to store and process the unwanted, unsaleable catch and bring it ashore to comply with regulations.

An alternative to landing is to document the catch using onboard monitoring. In recent years, some approaches have been evaluated to accurately document fish discards, including observer programs and remote electronic monitoring systems [3]. Both approaches require a significant amount of human resources to review and register the catch composition, which makes their application unfeasible on a large scale if full coverage is the goal [4]. To be able to handle the amount of information, several computer vision algorithms have been proposed to automate the detection, counting, and weighing of fish using images as input, e.g., [5]. Despite the promising results achieved by these approaches, all of them were trained with samples of all fish species expected to be present during the testing phase. This kind of setup is known as a closed-set problem. However, in real-world applications, certainly in fish catch monitoring, samples of unknown species are likely to appear during use of the system. For example, fish species not present in training datasets could be found in different fishing regions and during different seasons. These new species must be correctly handled by computer vision methods in order to avoid misclassification, which could negatively impact the recorded results for each species.

**Figure 1:**
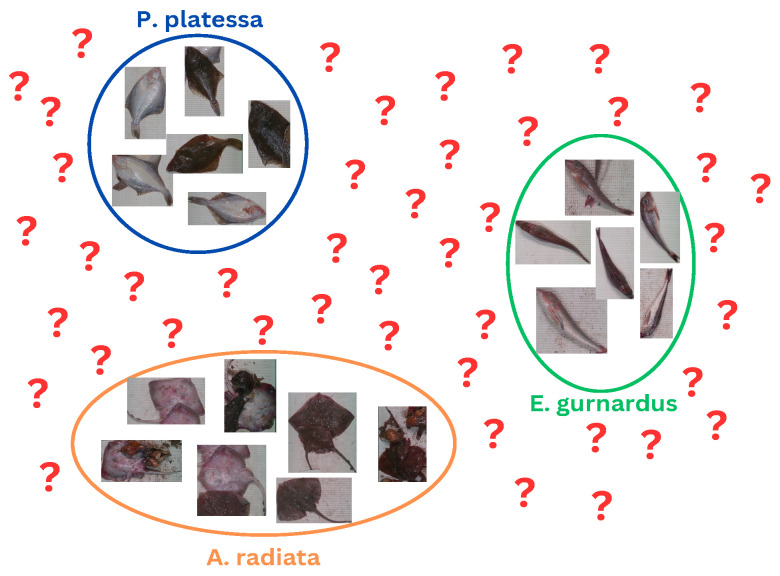
Open-set setup. Unseen species (?) may appear in the future and need to be recognized as unknown.

A situation where new, unknown classes might occur is referred to as an open-set recognition problem [6]. In fact, most real-life problems should be considered as open set. Unlike common closed-set problems, in open-set recognition, a classifier must be able to assign either one of the known classes or the unknown class. In this scenario, methods should be capable of deciding whether a new sample is too far from the training samples, in which case, it should classify it as unknown. Figure 1 shows an illustration of an open-set problem with three known species and samples from unknown species represented with question marks (?). In this example, the classifier defines boundaries based on the set of known species used during training. In a closed-set setup, new samples outside of the training distribution will be classified as one of the known species. However, there is a high likelihood that this is erroneous and, instead, these samples should be identified as unknown.

Over the last years, open-set methods have been evaluated in several applications, such as attributing photos to specific cameras [7], distinguishing between known and unknown sound events [8], open-set gesture-based verification for biometrics [9], human activity recognition [10], human–object interaction [11], and person re-identification [12], among others. Different types of approaches have been proposed for open-set recognition, including support vector machines [13], deep neural networks [14,15], generative adversarial networks [16], Gaussian-based approaches [17,18], and methods based on k-nearest neighbors [19]. Nevertheless, most of these approaches have been evaluated on datasets, such as ImageNet [20], CIFAR [21], and MNIST [22], which consist of classes with instances that are relatively easy to differentiate, though they can be challenging due to the quality of the images in some cases.

Unlike previous works, this paper focuses on the application and evaluation of open-set recognition for catch registration. Based on the existing literature, no studies have investigated the use of open-set recognition for automated fish catch registration. In contrast to the benchmarks used in the computer vision field to assess the performance of open-set methods, this study focuses on a real-world challenging scenario in the fisheries domain. In fisheries, distinguishing between different fish species is challenging because on the one hand, some species share high similarities in shape, size, and color, while on the other hand, within species, there is high variation in the appearance of the fish. In other words, the within-class variance is large, while the between-class variance can be low. In addition, on the conveyor belt, fish can be partially occluded by debris or even other fish instances, which makes the task even more complex. For this, the publicly available Fish Detection and Weight Estimation (FDWE) dataset [5] is used, which contains images from fish discards consisting of nine different species with a total of 2216 fish instances, including challenging scenarios with similar-looking species, within-species variation, and occlusions. Three state-of-the-art open-set methods are compared: OSNN [19], PISVM [13], and MGPL [18], which demonstrated their effectiveness in open-set scenarios. MGPL is a state-of-the-art parametric open-set classifier based on convolutional autoencoders and Gaussian prototypes, outperforming several successful open-set methods from the literature [18]. In addition, OSNN is a simple but powerful non-parametric method based on k-nearest neighbors [19], whereas PISVM [13] is based on SVMs and Weibull distributions, and it is one of the best SVM-based open-set methods. By evaluating MGPL, OSNN, and PISVM, the findings of this work can contribute to the research community, as the three methods cover distinct approaches, from simple ones such as k-nearest neighbors to more complex methods like convolutional neural networks.

The contributions of this paper can be summarized as follows:

- Comparison of three open-set methods in a real-world fish catch application.
- Proposal of a methodology for thorough evaluation of open-set methods using a cross-validation-like method on the FDWE dataset.
- Assessing the impact of new species appearing during testing in the process of fish catch registration.

## 2. Materials and Methods

### 2.1. Dataset

**Figure 2:**
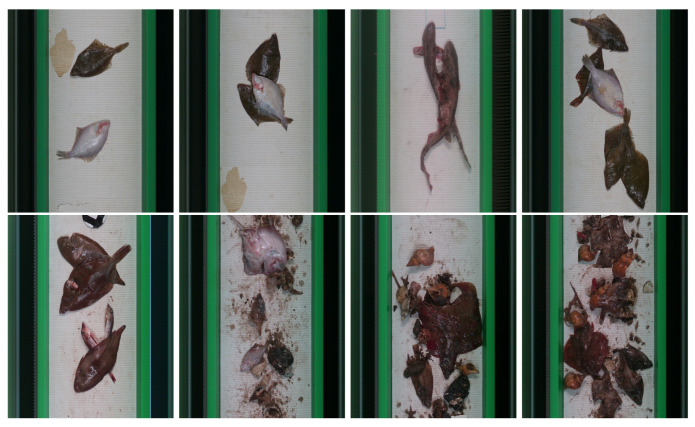
Images from FDWE [5] dataset.

In this work, the publicly available FDWE dataset [5] was used. This dataset contains images of catch, typical from demersal beam trawlers, filmed on a conveyor belt. The FDWE dataset has 1086 images with a total of 2216 instances depicting nine common species from the North Sea. Per fish, the ground truth of this dataset contains: species, bounding box coordinates, weight in kilograms, and level of occlusion. In the case of occlusion, four levels were defined in this dataset: 0% (fully visible), 1–30%, 31–60%, and 61–90%, which are produced by either overlapped fish or debris. Figure 2 depicts some visual examples of this dataset including different levels of complexity ranging from fully visible fish to images where multiple fish from different species are present and partially occluded by debris.

The FDWE dataset is composed of training, validation, and test sets with 1595, 424, and 197 fish instances, respectively. Table 1 lists the original partitions of FDWE along with the species and number of instances in each partition. As shown in Table 1, this is an imbalanced dataset where more than 50% of the fish correspond to the majority class *P. platessa*, whereas species such as *S. rhombus*, *A. radiata*, and *S. canicula* have fewer than 60 fish instances each in the whole dataset. More details about this dataset can be found in [5].

Although this dataset has its own predefined partitions, the following sections will describe how the dataset was organized to create an open-set setup.

### 2.2. Multiple Gaussian Prototype Learning

To handle samples from unknown classes, Liu et al. [18] proposed MGPL. The MGPL method focuses on creating a latent feature representation in which known and unknown species can be separable, arguing that the complex distribution of known classes can be captured by multiple Gaussian prototypes. This makes the task of distinguishing between known and unknown species more suitable during the testing phase. The distribution of each class is captured by *K* prototypes, producing a total of K×C prototypes, with *C* being the number of known classes. Each Gaussian prototype is represented by three values: the mean, deviation, and class.

Using a conditional variational auto-encoder, the latent feature space is learned through both generative and discriminative processes. The generative process has the goal of creating a latent space where the latent features of instances from the same class should be projected around their corresponding Gaussian prototypes, while the discriminative process seeks to maximize the separation between prototypes of different classes and bring the prototypes of the same class closer together. The distance between Gaussian prototypes and latent features is computed using the Kullback–Leibler (KL) divergence. During inference, if the distance from the latent features of a new sample to its closest prototype exceeds a predefined threshold, the sample is predicted to be unknown; otherwise, it is classified as the same class as its closest prototype. For more detailed information about this method, refer to [18].

### 2.3. Probability of Inclusion SVM

Support vector machines (SVMs) have been widely used for closed-set classification problems in different fields [23,24]. In addition, adaptations of SVMs have been proposed for open-set problems based on statistical modeling of extreme values [13,25]. Among the best-performing SVM-based open-set methods, Jain et al. [13] proposed probability of inclusion SVM (PISVM) as an extension of the SVMs for open-set recognition. SVMs do not directly estimate probabilities; the predictions are based on the margin score, which refers to the distance between the sample and the hyperplane that represents the decision boundary. In contrast, PISVM extends the capability of SVMs introducing a probability model to reject samples from unknown species.

The PISVM approach trains binary classifiers using the one-vs-all approach [26]. Then, to model the probability of inclusion, a Weibull distribution is fitted for each of the known classes using the margin scores of the samples most confidently classified. At inference time, given a new sample, PISVM chooses the maximum probability of inclusion among all normalized probabilities produced by the binary classifiers. If the maximum probability is below a given threshold, the new sample is predicted as unknown, otherwise the class with the highest probability will be assigned to the new sample. For more detailed information on PISVM, the reader may refer to [13].

### 2.4. Open-Set Nearest Neighbor

The OSNN algorithm [19] is a simple and powerful method to deal with open-set scenarios and it is based on the traditional k-nearest neighbor (KNN). Unlike other methods, which apply a threshold directly to the similarity scores, OSNN applies a threshold to the distance ratio of the two most similar classes instead. As presented in [19], compared to other open-set methods [6], the use of a ratio-based threshold allows the boundaries of the known classes to be limited in a better way, minimizing the error of the unbounded open space. Given a new sample *p*, the distance ratio *R* is computed using Equation (1)

(1) $$R=\frac{d(p,q)}{d(p,s)}$$

where *q* and *s* are the first and second closest samples of *p* from the training set, respectively, and *d* refers to a distance metric in the feature space. The two closest samples *q* and *s* must belong to different classes. In the case of the ratio, if *R* is greater than the predefined threshold, *p* will be rejected as unknown, otherwise *p* will be classified as the same class of its closest sample (*q*). Moreover, the versatility of OSNN allows the use of different distance metrics and different kinds of features as input. For a more detailed description of OSNN, the reader may refer to [19].

## 3. Experimental Setup

Next, the partitions used to simulate an open-set scenario are presented in Section 3.1, the training protocols for the evaluated open-set methods are detailed in Section 3.2, and the metrics used to compare the approaches are described in Section 3.3.

### 3.1. Dataset Partitioning

In this work, the performance of classifiers in open-set setups is evaluated. Instead of using the complete original images from the FDWE dataset, where multiple fish instances may appear in the same image, crops were extracted using the bounding box coordinates from the annotations trying to isolate single fish in each crop and duplicated instances were removed.

The conducted experiments were inspired by open-set protocols used in previous studies [19,27]. To train and compare the performance of the open-set classifiers, five experiments were conducted using different species as part of the unknown class in the validation and test sets. For each experiment, three splits (training, validation, and test) were used to create the open-set setup. The three splits included fish instances from known species. Due to the uneven distribution of species in the dataset, from the nine species of the FDWE dataset, the four species with the highest number of fish were considered as known species in all the experiments. The remaining five species were treated as unknown, with two assigned to the validation set and three to the test set. The validation set contains a set of unknown species to optimize thresholds, whereas the test set also includes unknown species that are different from the ones used in the validation set, to assess and compare the final performance of the methods.

**Figure 3:**
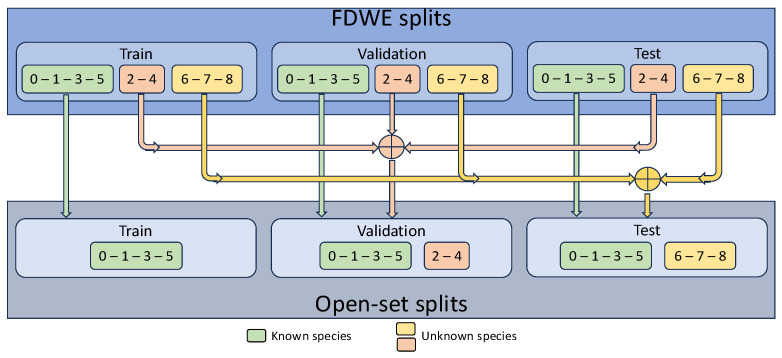
Example of the open-set partitioning process at species level.

The authors of FDWE [5] have already partitioned the dataset into training, validation, and test sets, ensuring that no fish instance appears in different partitions. Therefore, in this study, for the known species, the original FDWE partitions were used to avoid repetition of fish instances across partitions. Figure 3 illustrates an example of the partitioning process. As shown in the illustration, for the known species, the training, validation, and test sets in the open-set experiments were composed of instances extracted from the corresponding training, validation, and test sets of FDWE. Conversely, all fish instances of the unknown species from the three FDWE partitions were grouped together either in the validation or test set. It is important to mention that in the experiments, no distinction was made between the unknown species: all their samples were labeled as unknown.

The partitions used in each experiment are listed in Table 2. Species are represented by their corresponding indexes from the FDWE dataset: 0 for *P. platessa*, 1 for *S. solea*, 2 for *S. rhombus*, 3 for *E. gurnardus*, 4 for *S. maximus*, 5 for *L. limanda*, 6 for *A. radiata*, 7 for *M. merlangus*, and 8 for *S. canicula*. Additionally, the number of known and unknown instances per split are presented.

### 3.2. Training Protocols

For MGPL and PISVM, their original implementations were used. The code for the MPGL method is available at https://github.com/LiuJMzzZ/MGPL (accessed on 28 February 2025), whereas the code for PISVM can be found at https://github.com/ljain2/libsvm-openset (accessed on 28 February 2025).

In the case of MPGL, the model was trained on the known species for 100 epochs with a batch size of 16, using images of 224×224 as input, and a latent space of 512. For the remaining parameters, default values were used for training the encoder and decoder and defining the Gaussian prototypes of MGPL, including a learning rate of 0.001 , 3 prototypes per class, and Adam as the optimizer. To define the threshold for identifying unknown species, a grid search was performed on the validation set considering the binary F1-macro as the evaluation metric, where all instances were classified as either known or unknown. For the grid search, the distances from the instances in the validation set to their closest prototype were used as potential thresholds. The distance that resulted in the highest binary F1-macro was selected as the optimal threshold.

In the case of OSNN and PISVM, 512 features extracted by ResNet-18 [28] were used as input. The ResNet-18 model was trained on the known species for 100 epochs with a batch size of 16, using ImageNet weights as the starting point, input images of 224×224 , a learning rate of 0.001 , Adam as the optimizer, a weight decay of $5\times 10^{-5}$ , and cross-entropy loss with label smoothing of 0.1 . For data augmentation, horizontal flip, scaling, and color jitter were applied. Regarding PISVM, an SVM with radial basis function as the kernel with C=4 and γ=2.91 was used, and these values were optimized on the validation set. To determine the optimal threshold for both methods, a grid-search procedure was executed on the validation set testing values ranging from 0.01 to 1 with steps of 0.001 . The threshold that produced the best binary F1-macro was considered to be used on the test set. Table 3 presents the thresholds used per partition to evaluate the performance of the three methods on the test set.

### 3.3. Metrics

For evaluation, due to the imbalanced dataset, the F1-macro (Equation (2)) was used to compare the evaluated methods. The F1-macro is the average of all per species F1-scores (Equation (3)). In the equations, *C* refers to the total number of classes. For each class *i*, the precision (*P*) refers to the fraction of correctly classified fish instances among all fish predicted as class *i*, and recall (*R*) is the fraction of correctly classified fish instances among all fish belonging to class *i*.

(2) $$F1\mathrm{-macro}=\frac{1}{C}\sum_{i=1}^{C}{F1}_{i}$$

(3) $$F1=\frac{2\times P\times R}{P+R}$$

Additionally, to compare the performance of the evaluated classifiers in open-set conditions, the area under the receiver operating characteristic curve score (AUROC) [29] was used. The AUROC is a threshold-free metric that has been used in the literature [18,29,30] to compare the ability of methods to differentiate between known and unknown classes. This metric evaluates the overall performance of the open-set methods at various thresholds, considering the setup as a binary problem.

## 4. Results

In this section, the performance of the evaluated methods is assessed and compared. First, the performance of both methods is compared in a closed setup to assess their effectiveness when all species are known. Subsequently, their performance is evaluated in an open-set setup to measure the impact of unknown species that appear during testing.

### 4.1. Closed-Set Results

First, a closed-set experiment was conducted to assess the performance of the methods when no unseen species exist in the test set. For the closed-set setup, the methods were trained and evaluated on the same four known species. For closed-set inference of MGPL, the same strategy used in the work of Liu et al. [18] was applied. Specifically, the class of a new sample is given by the class of its closest prototype. In the case of OSNN, which is based on KNN, a KNN classifier with K =3 (value optimized on the validation set) was used for inference in this scenario. For PISVM, the class with the highest probability was directly assigned to the samples.

**Figure 4:**
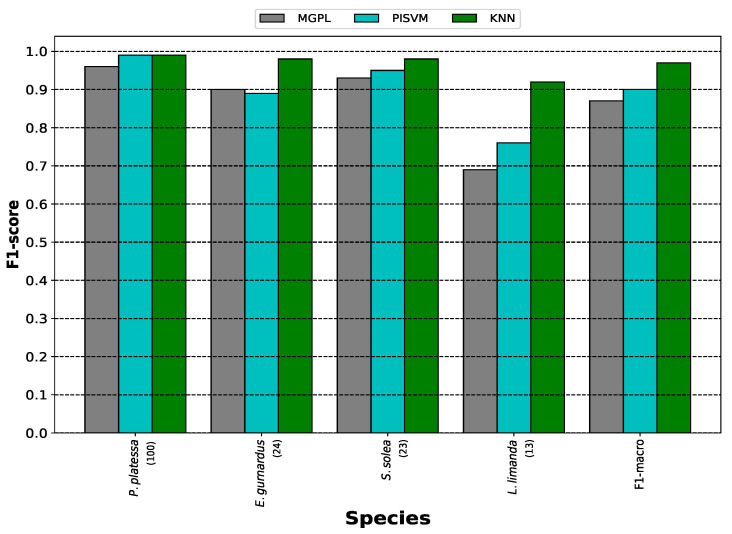
Closed-set results.

As shown in Figure 4, KNN outperformed MGPL and PISVM per species and in the overall F1-macro. The KNN algorithm achieved an F1-macro of 0.97 , improving the F1-macro of MGPL and PISVM by 0.10 and 0.07 , respectively. Per species, KNN reached an F1-score greater than or equal to 0.98 in three of the four species, except in the case of *L. limanda*, where its F1-score was 0.92 . The biggest difference in terms of F1-score between the three approaches occurred with *L. limanda*, where KNN outperformed MGPL by 0.23 and PISVM by 0.16 .

**Figure 5:**
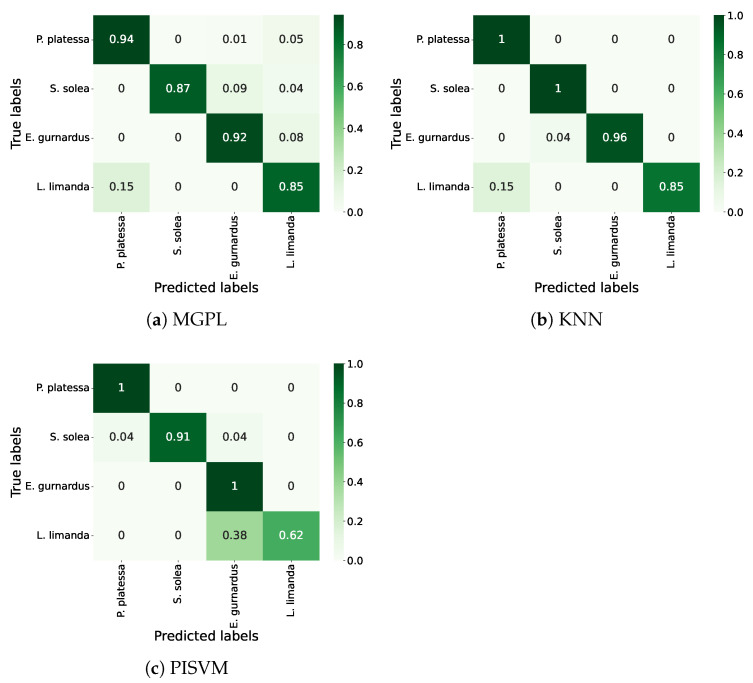
Confusion matrices in a closed-set setup.

Figure 5 presents the confusion matrices of the assessed methods. In total, MGPL misclassified 13 instances, whereas PISVM and KNN misclassified 7 and 3 fish, respectively. Considering the majority class, *P. platessa*, where all approaches obtained the highest F1-score, MGPL correctly predicted 94% of the instances, whereas PISVM and KNN correctly classified all instances.

On the other hand, considering *L. limanda*, the least representative species in the dataset, both MGPL and KNN misclassified 15% of the instances as *P. platessa*, whereas PISVM misclassified 38% of the instances as *E. gurnardus*. In addition, MGPL incorrectly classified five *P. platessa* and one *S.solea* as *L. limanda*, PISVM misclassified two instances of *S. solea*, one as *P. platessa* and the other as *E. gurnardus*, and KNN misclassified one *E. gurnardus* as *S. solea*.

### 4.2. Open-Set Results

**Figure 6:**
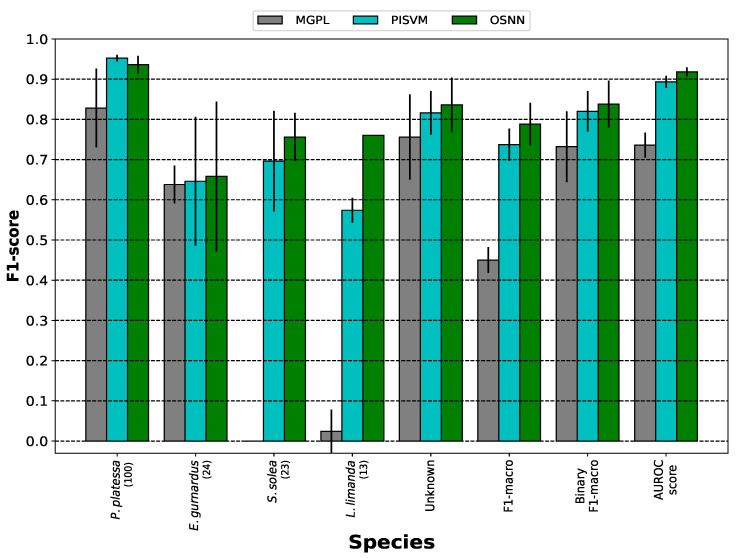
Open-set results.

To assess the ability of the methods to detect unknown fish species, five experiments were conducted using the partitions described in Section 3.1. The means and standard deviations from the five experiments are presented in Figure 6. In the case of the F1-macro, five classes were considered to compute this metric: the four known species and the unknown class. In contrast, for the binary F1-macro, two classes were considered: known and unknown. In the case of the known class, all four known species were encapsulated under this label. The binary F1-macro and the AUROC score were used to compare how well the approaches differentiate between known and unknown species as a binary classification problem. The former uses a fixed threshold, whereas the latter evaluates the performance of the methods across various thresholds.

Based on the five experiments, OSNN outperformed the other two methods reaching an overall F1-macro of 0.79±0.05 , surpassing the results achieved by MGPL and PISVM by 0.34 and 0.05 , respectively. Considering the binary metrics, OSNN and PISVM were the best-performing methods. The OSNN algorithm obtained a binary F1-macro of 0.84±0.06 followed closely by PISVM with a binary F1-macro of 0.82±0.05 . Moreover, OSNN and PISVM achieved AUROC scores of 0.92±0.01 and 0.89±0.02 , respectively. Conversely, MGPL showed limitations in distinguishing between known and unknown species, with an AUROC score of 0.74±0.03 . Based on the F1-macro, the repeated measures ANOVA along with post hoc pairwise t-tests with Bonferroni correction confirmed that OSNN and PISVM significantly outperformed MGPL. However, no significant difference was observed between them.

In the specific case of recognizing unknown species, MGPL achieved an F1-score of 0.76±0.11 , whereas OSNN and PISVM notably outperformed MGPL, achieving F1-scores of 0.84±0.07 and 0.82±0.05 , respectively. Considering the performance on the known species, OSNN was the best-performing approach in three out of four species, only surpassed by PISVM on the majority class, *P. platessa*. As expected, most of the incorrectly classified instances in the closed-set configuration, where the classifiers were less confident, were classified as unknown in the open-set setup. By comparing the open-set results of the three methods with the closed-set setup, it can be observed how the introduction of new species in the test set affects the performance of both classifiers. Compared to the closed-set setup, the F1-score of OSNN dropped by 0.05 , 0.32 , 0.22 , and 0.16 when evaluated on *P. platessa*, *E. gurnardus*, *S. solea*, and *L. limanda*, respectively. A similar trend was reported for PISVM, where the F1-score decreased by 0.04 on *P. platessa*, 0.24 on *E. gurnardus*, 0.25 on *S. solea*, and 0.19 on *L. limanda*. Conversely, MGPL had a drastic performance drop, especially on *L. limanda* with a drop of 0.67 and on *S. solea* where all its instances were classified as unknown.

Considering the best-performing method, in the five experiments, OSNN correctly classified most of the *P. platessa* instances. Out of 100 instances per experiment, the maximum number of misclassified fish was 4 resulting in an F1-score of 0.936±0.03 . All misclassified instances were predicted as unknown. For the 24 instances of *E. gurnardus*, OSNN achieved an F1-score of 0.658±0.17 , misclassifying only 3 instances as part of the unknown class. Among the 13 instances of *L. limanda*, 5 instances were misclassified as unknown, reaching an F1-score of 0.76±0 . Concerning *S. solea*, OSNN correctly classified between 14 and 17 instances, and 6 to 9 instances were predicted as unknown, obtaining an F1-score of 0.76±0.06 . Finally, regarding the samples of the unknown species, OSNN achieved an F1-score of 0.84±0.06 . In most cases, OSNN confused most of the unknown fish instances with *P. platessa* and *E. gurnardus*.

**Figure 7:**
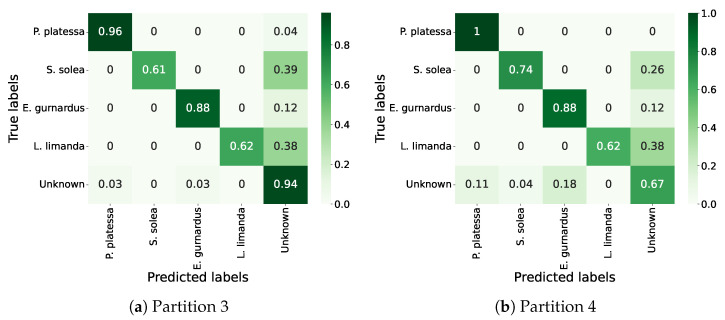
Confusion matrices of OSNN in an open-set setup.

Figure 7 presents the confusion matrices corresponding to the highest and lowest performance of OSNN on the five partitions. This method achieved its best performance when evaluated on partition 3 with an F1-macro of 0.84 and an AUROC score of 0.93 . In this experiment, instances of *S. maximus*, *A. radiata*, and *S. canicula* were used as unknowns in the test set. The achieved F1-macro can be explained by the visual dissimilarities between the known and unknown species in this partition, allowing the classifier to differentiate between known and unknown species more effectively. Among the unknown species, OSNN incorrectly classified 6% of the unknown instances, 3% as *P. platessa* and 3% as *E. gurnardus*.

On the other hand, its lowest result was obtained on partition 4, where the F1-macro was 0.72 with an AUROC score of 0.91 . In this partition, instances of *S. rhombus*, *A. radiata*, and *M. merlangus* were used as unknowns in the test set. Only *A. radiata* looks completely different from the known species, whereas *S. rhombus* and *M. merlangus* share visual similarities with them. In this experiment, OSNN erroneously classified 33% of the unknown instances, 11% as *P. platessa*, 4% as *S. solea*, and 18% as *E. gurnardus*. Of the 35 instances predicted as *E. gurnardus*, all were instances of *M. merlangus*, a species quite similar to *E. gurnardus*. Among the seven instances predicted as *S. solea*, all were instances of *S. rhombus*, which has a different shape compared to *S. solea*, but their colors share some similarities. Finally, of the 21 instances from the unknown species classified as *P. platessa*, some were instances of *S. rhombus*, which share some similarities with *P. platessa*. In addition, the seven *S. rhombus* instances were in the ventral side-up position. Additionally, 62% of the incorrectly classified instances were from *A. radiata*, a species with a shape entirely different from *P. platessa*, though they share some color similarities. Among those instances, 46% were in a ventral side-up position. The incorrect classifications can be explained by the class imbalance in the dataset, where *P. platessa* is represented by a large number of instances placed in a ventral side-up position.

**Figure 8:**
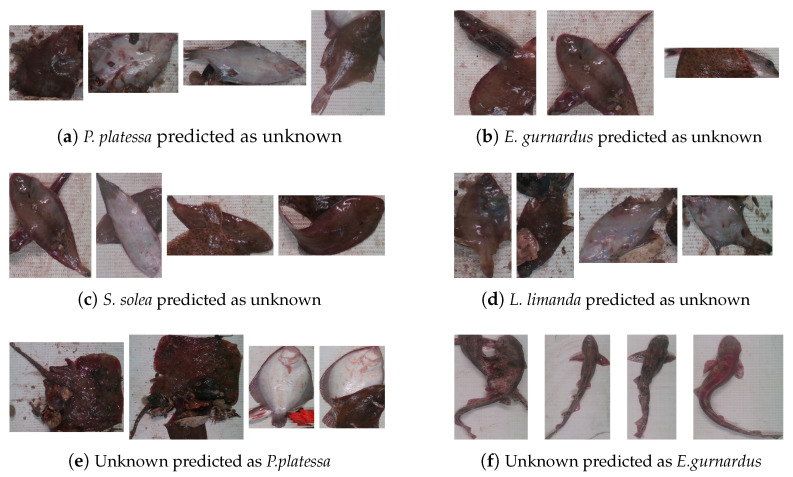
Incorrect predictions on partition 3.

**Figure 9:**
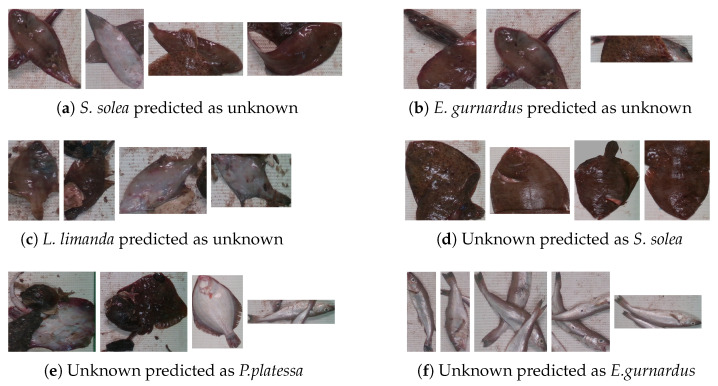
Incorrect predictions on partition 4.

Figure 8 and Figure 9 present some incorrectly classified instances on partitions 3 and 4. As these figures show, some known instances were misclassified as part of the unknown class. In the case of *S. solea* and *E.gurnardus*, incorrect predictions can be attributed to partially occluded fish caused by the presence of more than one fish, even from different species, in the same image. As observed, one of the main factors that contributes to the misclassification of instances of *E. gurnardus* as unknown is the higher degree of occlusion. Conversely, most instances of *S. solea* are not occluded; however, the presence of more than one fish in the image increases the uncertainty of the classifier, leading to misclassification as unknown. Moreover, the incorrect prediction of instances of *P. platessa* and *L. limanda* can be explained by the presence of debris that introduces noise, increasing the uncertainty of the classifiers.

With respect to instances from the unknown species that were assigned to one of the known species, some instances were confused because of visual similarities. Due to this fact, instances of the unknown class *M. merlangus* were confused with the known class *E. gurnardus*. In contrast, some unknown instances that look entirely different from the known classes were incorrectly classified as part of the majority class, particularly when fish were placed in a ventral side-up position. The majority class includes several instances placed with their ventral side up in the training set. Furthermore, instances of the unknown class *S. canicula* were classified as part of the known class *E. gurnardus*. Despite the clear visual differences between these species, OSNN was not able to differentiate between them. One possible explanation is that the features extracted by ResNet-18 need to better describe specific features of the different known samples. To overcome this limitation, strategies to include some samples from unknown species during training can be explored to enhance the feature representation.

## 5. Discussion

This study assessed the impact of the appearance of new species during the testing stage comparing the performance of three open-set recognition methods in the context of fish catch registration. The results highlight both the potential and limitations of the assessed methods in this domain. Unlike other domains, where open-set methods are typically evaluated on datasets containing classes that are relatively easy to differentiate, real-world fish classification is a complex scenario that presents additional challenges, such as imbalanced datasets, strong similarities between different fish species, and even occlusion caused by debris or other fish.

Experiments demonstrated the superiority of OSNN [19] and PISVM [13] compared to MGPL [18] in the context of fisheries, both detecting known and unknown species. OSNN outperformed PISVM achieving an F1-macro of 0.97 when evaluated only on known species. However, its performance dropped when new species were introduced in the test set, achieving an F1-macro of 0.79±0.05 . Furthermore, the superiority of OSNN in the open-set fish classification challenge was also demonstrated by the AUROC score of 0.92 , improving the AUROC scores of PISVM and MGPL by about 3.00 and 18.20 percentage points. In some cases, species with very similar shapes, sizes, or colors were incorrectly classified. For example, when *M. merlangus* was used as an unknown class, the instances from this species were confused with one of the known species, *E. gurnardus*. In contrast, due to the imbalanced nature of the FDWE dataset, some unknown species with shapes completely different from the known classes were misclassified as part of the majority class. Most of those instances were placed ventral side up, which suggests that the model may be biased towards the majority class, as it is represented by several ventral side-up instances. This resulted in the misclassification of instances of unknown species such as *P. platessa*, given the distinctive white color of their ventral side and the predominance of *P. platessa* instances with this characteristic in the training set.

In the case of MPGL, one of its limitations could be related to the deviation used to define its Gaussian prototypes. Since the deviation of the Gaussian prototypes is not a trainable parameter and an identity matrix is used, all distributions belonging to different classes share the same deviation, which limits the definition of more effective decision boundaries for each prototype. Allowing more flexibility for this parameter could enhance the performance of this method. In the case of OSNN, the main limitation of this method is the use of the entire training set to compute distances during inference, which limits its efficiency in both inference speed and storage for large datasets. However, indexing schemes could be explored to improve the efficiency of this method.

Open-set methods have been evaluated on various datasets, making it difficult to have a direct comparison with the results reported in this study. Despite the limitations, the results reported in this paper are compared with other studies from the literature [31,32]. There are only a few studies evaluating the performance of open-set methods in fish classification. WildFishNet was proposed for wild open-set fish recognition by Zhang et al. [31], using the dataset introduced in [33] to evaluate its performance. WildFishNet achieved an accuracy of 0.846 among known species and an F1-score of 0.878 on instances from unknown species, using 685 species as known out of 1000 species. Using the same dataset, Akhtarshenas and Toosi [32] evaluated the use of an autoencoder to deal with unseen species. In their best configuration, their model reached an accuracy of 0.937 and 0.816 when 300 and 700 species were used for training, respectively. These results are comparable with the ones obtained in this study, where OSNN achieved an accuracy of 0.893±2.18 on the known samples and an F1-score of 0.836±0.07 on the unknown species. However, in the present study, these results were derived from five experiments, providing more insights into the impact of introducing variation among different unknown species as part of the test set. Despite some similar visual challenges with the previous studies, such as visually similar instances from different species, the process of registering fish catch is further complicated by the presence of several fish partially occluded by others or by debris, as well as the presence of more than one species per image in some cases.

Currently, several ongoing projects aim to enhance sustainability and compliance with regulations in fisheries through the use of machine learning-based systems, such as automating the fish catch registration process. However, to date, these systems have been designed for closed-set setups. In the process of registering fish catch, using a closed-set approach would result in unseen species being incorrectly classified as one of the known species with a negative impact on the counting report. As demonstrated in this study, the inclusion of unseen species affects the performance of closed-set methods; however, open-set recognition methods (OSNN and PISVM) are able to distinguish known from unknown species, reducing misclassification errors; hence, the miscounting impact of unseen species can be minimized. This functionality to recognize unknowns is crucial to achieve a more accurate species identification and minimize errors in the counts per species.

The results reported in this work evidenced that the presence of unseen species during the deployment poses challenges that must be addressed in order to use machine learning systems for real-world fisheries applications. In the experiments, most of the errors correspond to samples from known species being predicted as unknown. This type of error should be minimized; however, misclassifying a sample from a known species as an unknown is not a major issue. While it does require some human intervention to address the misclassification of unknowns, it does not lead to an error in the overall count of known species, as the instance is initially classified as unknown and then, after human review, it can be classified as the correct species. However, the reverse situation presents a challenge for open- and, especially, closed-set methods, with the latter lacking any mechanism for filtering unknown species. This type of misclassification results in a direct miscount, as the unknown species would be erroneously included in the count of the known species. Moreover, as the number of unseen species increases, it becomes more difficult for the classifiers to properly distinguish between known and unknown species [6,32].

Based on the challenges encountered in this study, future work will focus on approaches to overcome the occlusion problem and the presence of multiple fish in the same image, which increases the uncertainty of the classifiers. For this, segmentation approaches can be explored to reduce the negative impact of these scenarios. Moreover, methods need to be investigated to better describe the distributions of species, allowing for better separation between the known classes and the unknown class. For instance, strategies for combining features extracted from different types of descriptors to better differentiate between similar species can be tested to improve the performance of the evaluated methods. In addition, strategies such as active learning [34] should be explored to add a human into the learning loop, who can provide labels to the unknown species, to update the initial classification models. This allows the continuous labeling of new instances from unknown species and their inclusion in the training set, reducing continuously the open space risk. Moreover, hybrid methods that combine supervised learning with semi-supervised or self-supervised learning might help to improve the performance when dealing with unseen species, increasing the generalization ability of the methods. Additionally, given the vast amount of data gathered in this field, open-set methods could be useful for selecting relevant images where classifiers are more uncertain, allowing the models to be trained with less but more relevant data, thereby improving the performance and reducing the number of annotated images. Addressing these challenges through improved data collection, model refinement, and the integration of advanced learning techniques will be crucial in moving towards more reliable and scalable fish monitoring systems in fisheries.

## 6. Conclusions

This study assessed the impact of unseen species in fish catch registration systems comparing three open-set image-based methods for classification of known and unknown species. Results demonstrated that the inclusion of unseen species during deployment significantly affects the model performance of closed-set methods, but open-set methods could reduce the impact of these issues. Among the evaluated methods, the statistical analysis confirmed that OSNN and PISVM outperformed MGPL with significant differences. In terms of F1-macro, OSNN achieved an F1-macro of 0.79±0.05 , whereas PISVM reached an F1-macro of 0.73±0.04 . Additionally, the AUROC scores confirmed the superiority of OSNN and PISVM, with OSNN achieving the highest AUROC of 0.92 , followed by PISVM with 0.89 and MGPL with 0.74 . These results indicate that OSNN and PISVM are more effective at distinguishing between known and unknown species.

Experiments showed the potential of using open-set methods to distinguish unseen species from known species, thereby reducing the misclassification impact inherent in closed-set methods when new species are introduced during testing. This could benefit the catch registration process by reducing the number of miscounted instances when new species appear. The experimental results revealed that occlusion, the presence of debris or multiple fish in the same image, impact negatively the classification performance, increasing uncertainty and leading to misclassifications. The performance of open-set methods can be further improved in future work by incorporating instance segmentation approaches and focusing on challenges such as class imbalance, partially visible fish, class similarities, and intra-class variations.

The use of open-set methods as part of systems for automatic fish catch registration is an important step towards the deployment of those systems on fishing fleets at a large scale to properly handle the presence of new species in new fishing regions or different seasons. Future work needs to deal with solutions to then process the instances from unknown classes, for instance, in an active learning paradigm, with a human in the loop to label the instances with the new species and retraining of the classifier [34]. Overall, this research highlights the potential for open-set methods to enhance the scalability and effectiveness of fish catch monitoring systems, contributing to more sustainable fisheries management and reducing the negative impact posed by the appearance of unseen species during deployment. 

## Figures and Tables

**Table 1:** Number of fishes in the original partitions of FDWE dataset [5].

Species	Training	Validation	Test	Total
*Pleuronectes platessa*	876	206	101	1183
*Eutrigla gurnardus*	224	61	25	310
*Solea solea*	170	49	23	242
*Limanda limanda*	122	35	15	172
*Merlangius merlangus*	59	20	8	87
*Scophthalmus rhombus*	49	7	3	59
*Amblyraja radiata*	40	13	6	59
*Scophthalmus maximus*	35	28	11	74
*Scyliorhins canicula*	20	5	5	30
	1595	424	197	2216

**Table 2:** Partitions used in this study. In parenthesis, the amount of instances.

	Species Index				
	**Training**	**Validation**		**Test**	
**#**	**Known**	**Known**	**Unknown**	**Known**	**Unknown**
1	0–1–3–5 (1391)	0–1–3–5 (340)	2–4 (131)	0–1–3–5 (160)	6–7–8 (163)
2			6–8 (83)		2–4–7 (211)
3			2–7 (139)		4–6–8 (155)
4			4–8 (102)		2–6–7 (192)
5			6–7 (133)		2–4–8 (161)

**Table 3:** Thresholds used per partition.

	Thresholds		
**#**	**MGPL**	**PISVM**	**OSNN**
1	125.249	0.509	0.626
2	171.246	0.397	0.626
3	137.516	0.577	0.491
4	125.249	0.618	0.656
5	139.734	0.577	0.491

## Data Availability

Publicly available datasets were analyzed in this study. This data can be found here: https://dx.doi.org/10.4121/a6d5a40e-0358-47cf-9ec1-335df0e4a3c3 (accessed on 1 November 2024).
